# Decreased Risk of Osteoporosis Incident in Subjects Receiving Chinese Herbal Medicine for *Sjögren syndrome* Treatment: A Retrospective Cohort Study with a Nested Case-Control Analysis

**DOI:** 10.3390/ph17060745

**Published:** 2024-06-06

**Authors:** Chieh-Tsung Yen, Hanoch Livneh, Hua-Lung Huang, Ming-Chi Lu, Wei-Jen Chen, Tzung-Yi Tsai

**Affiliations:** 1 Department of Neurology, Dalin Tzu Chi Hospital, Buddhist Tzu Chi Medical Foundation, Chiayi 62247, Taiwan; 2 Rehabilitation Counseling Program, Portland State University, Portland, OR 97207-0751, USA; 3 Department of Rehabilitation, Dalin Tzu Chi Hospital, Buddhist Tzu Chi Medical Foundation, Chiayi 62247, Taiwan; 4 School of Medicine, Tzu Chi University, Hualien 97004, Taiwan; 5 Division of Allergy, Immunology and Rheumatology, Dalin Tzu Chi Hospital, Buddhist Tzu Chi Medical Foundation, Chiayi 62247, Taiwan; 6 Department of Chinese Medicine, Dalin Tzuchi Hospital, Buddhist Tzu Chi Medical Foundation, Chiayi 62247, Taiwan; 7 Graduate Institute of Sports Science, National Taiwan Sport University, Taoyuan 333325, Taiwan; 8 School of Post-Baccalaureate Chinese Medicine, Tzu Chi University, Hualien 97004, Taiwan; 9 Center of Sports Medicine, Dalin Tzu Chi Hospital, Buddhist Tzu Chi Medical Foundation, Chiayi 62247, Taiwan; 10 Department of Medical Research, Dalin Tzu Chi Hospital, Buddhist Tzu Chi Medical Foundation, Chiayi 62247, Taiwan; 11 Department of Environmental and Occupational Health, College of Medicine, National Cheng Kung University, Tainan 70428, Taiwan

**Keywords:** autoimmune disease, bone function, Chinese herbal medicine, nested case-control study

## Abstract

Sjögren syndrome (SS) is a long-lasting inflammatory autoimmune disease that may cause diverse manifestations, particularly osteoporosis. Though usage of Chinese herbal medicine (CHM) can safely manage autoimmune disease and treatment-related symptoms, the relation between CHM use and osteoporosis risk in SS persons is not yet recognized. With that in mind, this population-level nested case-control study aimed to compare the risk of osteoporosis with and without CHM use. Potential subjects aged 20–70 years, diagnosed with SS between 2001 and 2010, were retrieved from a national health claims database. Those diagnosed with osteoporosis after SS were identified and randomly matched to those without osteoporosis. We capitalize on the conditional logistic regression to estimate osteoporosis risk following CHM use. A total of 1240 osteoporosis cases were detected and randomly matched to 1240 controls at a ratio of 1:1. Those receiving conventional care plus CHM had a substantially lower chance of osteoporosis than those without CHM. Prolonged use of CHM, especially for one year or more, markedly dwindled sequent osteoporosis risk by 71%. Integrating CHM into standard care may favor the improvement of bone function, but further well-designed randomized controlled trials to investigate the possible mechanism are needed.

## 1. Introduction

Chronic inflammatory diseases, conditions of exaggerated defense responses to noxious mediators, are known to cause significant disability and mortality worldwide. Take the Sjogren syndrome [SS] as an instance. It is a chronic autoimmune disease featured by inflammation of the exocrine glands, with predominance of female patients [1]. A recent meta-analysis showed the incidence of physician-diagnosed SS is between 3.9 and 11.7 per 100,000 persons/year, with a pooled incidence of 6.9 [2]. Notably, SS has become more prevalent over time globally, creating a heavy financial burden for the healthcare system [1]. In the United States, analysis of 12,717 matched pairs of SS and non-SS persons between 2003 and 2013 showed that the annual medical cost owing to SS and its symptoms averages 14,387 US dollars (US$) for each SS patient, and more than twice that of those without SS ($6534) [3].

Besides the large health and social burden, recent evidence highlights that the persistent systemic inflammation associated with SS may incite the risk of extra-glandular manifestations, particularly in the development of osteoporosis [4]. Although the etiology of SS is complex and yet incompletely understood, the enhanced stimulation of inflammatory cytokines from T helper (Th) cells is presumed to be involved in the pathogenesis of SS [2,5]. Because of a dysfunctional immune system, some serum pro-inflammatory cytokines, such as interluklin-6 (IL-6), tumor necrosis factor (TNF)-alpha, as well as IL-17, are predominantly released from Th1 cells among those with SS [5,6]. The release of these mediators may favor the development of cell-mediated immunity, apoptosis of osteoblast cells, and deterioration of bone tissue, thus inciting risk of osteoporosis [7]. Some shared intracellular signaling pathways, like mitogen-activated protein kinases (MAPK) and receptor activator of nuclear factor κB (NF-κB) ligand (RANKL) pathway, have been assumed to link the crosswalk between inflammatory parameters and loss of bone density. [4,8]. One recent cohort study found that, compared to those without SS, subjects with SS who received standard treatment nearly doubled the likelihood of bone mass impairment [9]. In case of the emergence of osteoporosis, the death rate due to osteoporosis-related hip fracture would rise markedly and be comparable to some severe diseases, such as breast cancer and pancreatic cancer [10]. With that in mind, during the treatment of SS, personalized strategies for mitigating the likelihood of developing osteoporosis incident should be timely and judiciously implemented.

Recently, Chinese herbal medicine (CHM), a specialized therapy within traditional Chinese medicine, has been extensively employed in treating human diseases, especially in SS. A systematic review of 52 randomized control trials showed that CHM, delivered either alone or in combination with other Western medicine, may be more effective in managing primary SS symptoms than conventional medicine [11]. Chen and colleagues observed that some herbals, such as Gan-Lu-Yin and Jia-Wei-Xiao-Yao-San, have been implicated in controlling SS symptoms [12]. The mechanisms by which CHM aids those with SS may include regulation of inflammatory responses, increased antioxidant capacity, and improvement of sicca [13]. However, no studies have yet directly compared the therapeutic effect of CHM in delaying or minimizing the onset of osteoporosis in those with SS. Since existing treatments provide no effective cure for most autoimmune diseases, adding CHM to routine care might prevent or delay bone mass impairment. We, therefore, mapped out a nested case-control study to clarify whether adding CHM to the conventional treatment would reduce the chance of osteoporosis in those with SS.

## 2. Results

**Figure 1:**
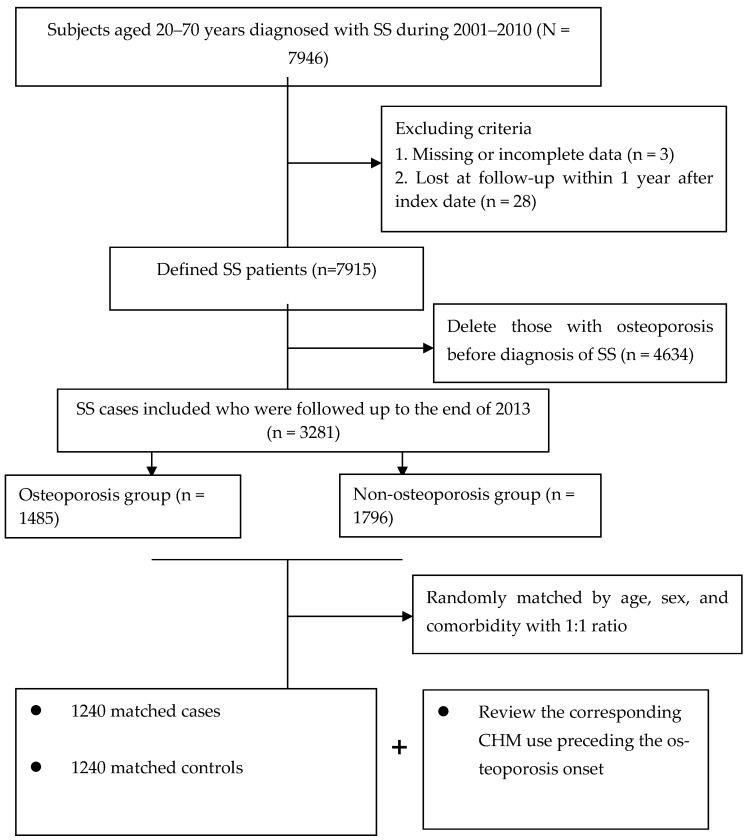
Flowchart of subject selection.

The final study cohort included 2480 patients (Figure 1). Throughout the study period, a total of 1240 matched pairs of SS subjects with and without osteoporosis were included. The mean age for both groups was 48.5 years. Also, over half of the enrollees lived in cities and were at the middle-income level. Following the random matching process, the two groups did not differ in initial demographic data, number of comorbidities or anti-osteoporotic medications. Details of the relevant variables at baseline are shown in Table 1.

During the study timeframe, 28.5% of the cases and 53.1% of the controls received CHM treatment. In the multivariate analysis of CHM use and osteoporosis risk, those with a history of CHM use had a 52% reduced likelihood of osteoporosis, in comparison with those who never used CHM (adjusted OR 0.47; 95% CI: 0.39–0.57). Table 2 shows that the participants in level 3 experienced a 71% reduced risk, which implies an exposure–response inverse relation (Table 2). Table 3 documents the beneficial impact of CHM stratified by age and gender. On the whole, this exposure–response relation between CHM use and reduced risk of osteoporosis appeared persistent within the cohort, regardless of the subject’s age and gender.

**Figure 2:**
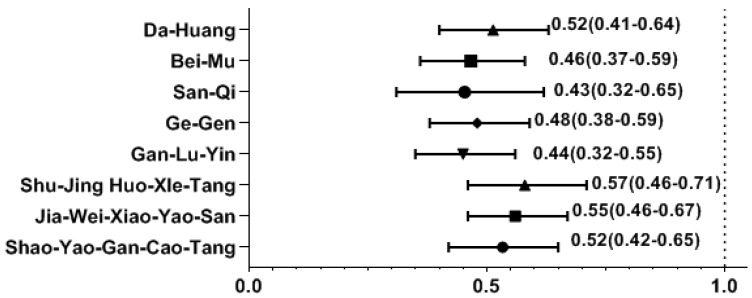
Effect of CHM on the risk of osteoporosis by multivariate forward conditional logistic regression. Y-axis: Chinese herbal medicines; X-axis: odds ratio.

Among the most common single-herb and multi-herb formulae used to treat SS, we found that the use of several CHM products was correlated with a decreased likelihood of osteoporosis. The osteoporosis risk was significantly reduced in those using CHM products containing Da-Huang, Bei-Mu, San-Qi, Ge-Gen, Shao-Yao-Gan-Cao-Tang (SYGCT), Gan-Lu-Yin (GLY), Jia-Wei-Xiao-Yao-San (JWXYS), and Shu-Jing-Huo-Xue-Tang (SJHXT) (Figure 2).

## 3. Discussion

Patients with SS have been recognized as having a higher predisposition for systemic impairment of bone mass as compared to health controls [9]. As no specific cure yet exists for SS, we should lay emphasize on the crosstalk between use of complementary and alternative therapy and the prevention of incident osteoporosis for those with SS. Herein, we used a population-based nested case-control study designed to cast light on this issue, and findings of our study revealed that patients with SS receiving CHM along with conventional care exhibited a substantially lower likelihood of developing osteoporosis. A longer duration of CHM treatment markedly reduced the likelihood of osteoporosis. Although the lack of similar studies for comparison makes confirmation of this result difficult, the current findings expand the literature that supports the benefit of CHM in treating chronic illnesses, especially for those with bone-related diseases [14,15].

We also found that females benefited more from CHM treatment than males. An expanding body of evidence on gender differences shows that, in general, females have more health-related knowledge and exhibit more healthy behaviors than males [16,17]. In this case, females may be more willing to conform to the prescribed medical regimen, which in turn lessens the subsequent risk of osteoporosis. Additionally, the release of sex hormones, particularly estrogen, may also partially explain this beneficial phenomenon. Accumulating evidence shows that estrogen can stimulate the activation and proliferation of satellite cells, which are required for the growth, maintenance, and regeneration of bone marrow [18,19]. Consequently, for women, the secretion of estrogen may potentially enhance the therapeutic impact of CHM in delaying or preventing the onset of osteoporosis.

We observed that the use of certain herbal products was tied to the reduction of osteoporosis incidents. Of the single-product formulae commonly used to treat SS, we noted that San-Qi, Ge-Gen, and Bei-Mu could lessen the risk of osteoporosis by more than 50%. The mechanisms whereby these herbals protected against the incidence of osteoporosis may primarily correlate with the regulation of inflammatory responses. In traditional Chinese medicine, San-Qi and Bei-Mu are frequently employed for relieving pain [20]. Several recent experimental studies documented that these herbs may act in a dose-dependent manner to reduce the levels of inflammatory factors involving IL-1, IL-6, and TNF-alpha in lipopolysaccharide-stimulated macrophage RAW264.7 cells via the suppression of NF-κB activation [20,21,22]. Not only is NF-κB the centerpiece of inflammatory responses, it is also involved in bone metabolism [8]. Upon activation, NF-κB is transferred into nuclei, where it can boost the mRNA expression levels of c-Fos and nuclear factor of activated T-cells, cytoplasmic 1 (NFATc1), which are both confirmed transcription factors that regulate osteoclast formation and function [23], thereby affecting the risk of osteoporosis.

Another herbal product proven effective in lessening the risk of osteoporosis is Da-Huang. One review figured that emodin, a major essence purified from this herb, has shown antitumor and anti-inflammatory effects through the inhibition of the matrix metallopeptidase gene via regulation of the various cellular NF-κB and MAPK pathways [24]. Both in vitro and in vivo studies have shown that modulation of the MAPK signaling pathway can minimize the expression of downstream targets required for osteoclast formation [25,26]. These mechanisms may account for the benefit of Da-Huang found in our subjects with SS.

Among the commonly used multi-herb products, some herb formulae were shown to decrease the risk of osteoporosis incidence. For example, use of SYGCT was associated with a nearly 50% lower risk of osteoporosis. This medication may rescue the decreased phosphorylation of glycogen synthase kinase 3 (GSK-3) in patients with chronic diseases [27]. Activation of GSK-3 can modulate the inflammatory response via downregulation of the PI3K-AKT-mTOR inflammasome pathway, an action proven to help regulate osteoclast precursor proliferation and apoptosis [28]. It is believed that high levels of osteoclastogenic cytokines may amplify the RANKL-induced osteoclastic bone resorption, thereby inciting, to some extent, the risk of bone mass loss [7,26].

Uses of JWXYS and SJHXT were found to diminish the risk of incident osteoporosis among SS participants. These remedies have been reported to appreciably decrease plasma levels of IL-6 and TNF-alpha via the suppression of the NF-κB pathway in both human and animal research [29,30]. In a prior murine model, it was revealed that rats fed 60 mg/kg daily of a natural compound from GLY for 20 days markedly modified RANKL-induced osteoclastogenesis in a dose-dependent manner through inhibiting the NFATc1and NF-κB luciferase activities [31]. Since deregulated NF-κB activation has been involved in various inflammatory diseases [8,32,33], targeting this intracellular pathway may be a treatment option in clinical practice, especially for patients with SS and subsequent bone mass impairments.

In this pioneering work, some noteworthy limitations must be acknowledged. First, our findings were derived from a retrospective analysis using the ICD-9-CM diagnosis codes. Misclassification errors may have arisen to propagate the biased estimates. Nevertheless, the NHI regularly reviews medical charts to verify the accuracy of claims and medical charge data. To further diminish the chance of misclassification in this work, we selected the cases with either SS or osteoporosis only after they were recorded as having at least two ambulatory or one inpatient claim reporting consistent diagnoses. At the same time, the SS code was further confirmed by CIR record. On this note, since any misclassification of cases in administrative data will occur randomly, if anything, such errors will cause the observed overall OR to be biased toward a more conservative estimate. Second, as all of the analytical data were extracted from a claims-based database, several key factors, such as lifestyle behaviors, body mass index, family history, and biochemical data, were not available. Thus, a study of larger cohorts of SS persons with a randomized clinical trial design is warranted to uncover the potential mechanisms of CHM use after adjusting for the possible confounders. Third, because no information on SS severity was available from the database, we managed to adjust for it by conducting two sensitivity analyses. First, we only recruited SS subjects who reported no comorbidities to reanalyze the relationship of interest. The re-analysis indicated that, as compared to SS patients without CHM use, the selected group of SS patients with CHM use still had a significantly lower incidence rate for osteoporosis, with adjusted OR of 0.50 (95% CI, 0.40–0.62). The second approach was that we merely retrieved enrollees if they ever received hospitalization treatment due to SS to be a surrogate variable for the severity of the disease, and the reanalysis yields essentially the same result (adjusted OR: 0.54; 95% CI = 0.43–0.68). These sensitivity analyses support the notion that disease severity was not likely to introduce a remarkable effect on our conclusion, and namely adding CHM to conventional therapy may reduce the subsequent risk of osteoporosis in SS patients.

## 4. Methods

### 4.1. Data Source

This study was approved by the facility’s institutional review board (No. B11004025-1). All population-level data used in this nested case-control study were collected from the nationwide database, which covers one million members randomly selected from National Health Insurance (NHI) in Taiwan [34]. With random extraction from all beneficiaries under the NHI program by age and sex, this database provides a representative sample of the Taiwanese population. The database included encrypted patient data that documented sex, date of birth, medical diagnoses in the form of the International Classification of Diseases, Ninth Revision, Clinical Modification (ICD-9-CM) codes, dates of hospital admission and discharge, dates of outpatient visits, medical procedures performed, and prescribed medications covered by the NHI, including CHM.

### 4.2. Underlying Cohort Establishment

In the beginning, a total of 7946 SS patients whose aged ranged from 20 to 70 years were selected with the principal ICD-9-CM code of 710.2 between 2001 and 2010. To correct for misclassification bias, all selected claims were further checked via the application of the catastrophic illness registry (CIR) record. Under the NHI program, those with autoimmune illnesses or malignancy can apply for the CIR card for exemption from copayment of all medical costs. The CIR application must be confirmed by experts and related treatments, and therefore the diagnosis can be considered highly accurate. The date of CIR approval due to SS was viewed as the cohort entry date. Finally, patients experiencing osteoporosis before cohort entry or those with incomplete demographic information were removed (n = 4665). The remaining participants were followed up until the earliest diagnosis of osteoporosis, death, or the end of 2013, whichever came first.

### 4.3. Ascertainment of Case and Control Groups

The major outcome was osteoporosis onset that occurred from 2002 to 2013, but after SS onset. Participants were defined as having osteoporosis if they received at least two outpatient clinic visits in one year or one hospitalization during the study timeframe, due to this condition (ICD-9-CM code 733.0) [35]. The first medical visit for osteoporosis treatment was viewed as the index date. Afterwards, each case was randomly matched to one control without osteoporosis according to age, sex, and number of comorbidities (Figure 1). The index date assigned to each control corresponded to the date of osteoporosis diagnosis of the matched case, thus ensuring identical observational probability for all recruited subjects within the study period.

### 4.4. Categorization of Chinese Herbal Medicine Use

The identification of exposure of CHM use is the record regarding visits to Chinese medicine practitioners that occurred from the cohort entry to the index date. In this exploration, CHM users were identified as those who received the CHM treatments for SS or its symptoms for more than 30 days. Amongst CHM users, they were further divided into three subgroups based on the days of CHM prescriptions within the study period: level 1 (31–180 days), level 2 (181–365 days), and level 3 (366 days or more). The manner that we adopted was to assess the exposure–response impact of CHM in the prevention of osteoporosis.

### 4.5. Definition of Covariates

Covariates considered contained sex, age, prior medical comorbidities, individual monthly salary, and urbanization of residential area. We first estimated the premium payment category as a proxy of monthly salary, and then transformed this to ordinal indicators in terms of the 25th, 50th and 75th percentile. A widely-adopted urban-rural classification in Taiwan was used to classify the urbanization of enrollees’ dwelling [36]. This indicator was created by considering several dimensions of urbanity, such as population density per square kilometer, proportion of persons with college qualifications or above, ratio of the population aged 65 years or older, proportion employed in agriculture, and number of clinicians per 100,000 inhabitants. Then we split the enrollees’ residences into urban, suburban and rural areas. Individual medical comorbidities were defined as a condition found at least once on an inpatient treatment record or twice on outpatient treatment records in the year preceding the cohort entry date. The Charlson–Deyo comorbidity index (CCI), an approach that took into account both the number and severity of 17 pre-defined comorbid conditions, was used to quantify participants’ burden from comorbidities [37]. Additionally, the prescription of anti-osteoporotic medications among enrollees was considered. Subjects, who took calcium supplements, vitamin D, calcitonin, bisphosphates, selective estrogen receptor modulators, sex hormones, strontium or RANKL inhibitor for more than six months after cohort entry, would be deemed as users of anti-osteoporotic medications.

### 4.6. Evaluation of Data

This study utilized SAS software for Windows version 9.3 (SAS Institute, Inc., Cary, NC, USA) to conduct the statistical analysis. Descriptive statistics, including mean, standard deviation (SD), frequency and percentages, were applied as appropriate. The discrepancy in each variable between CHM users and non-CHM users was determined by employing standardized mean differences. A standardized difference <0.1 indicated that the difference between the two groups was statistically insignificant [38]. The univariate and multivariate conditional logistic regressions were then used to calculate odds ratios (OR) and corresponding 95% confidence intervals (CI) for the relation between CHM use and subsequent osteoporosis incidence. We also conducted one stratified analysis, by age and gender, to test the robustness of the results. We used multiple forward stepwise conditional logistic regression to identify which commonly prescribed CHM products may diminish osteoporosis risk. All inferential analyses were two-tailed with an alpha level less than 0.05.

## 5. Conclusions

Faced with the dire harmful manifestations caused by intrinsic inflammation, this study goes beyond traditional therapy thinking to explore the impact of CHM use on the prevention of osteoporosis among SS patients. We noted that integrating CHM into conventional care may lower subsequent risk of developing osteoporosis by 52%. On top of that, this therapeutic impact appears to be dose-dependent, with more intensive CHM treatment providing a greater reduction in osteoporosis. Findings from the present study may be a reference for integrating CHM into routine disease management for those with rheumatological disorders. Another merit of this study is to provide encouragement for further research on the adoption of CHM products, as well as in vivo studies, to explore the beneficial mechanisms of CHM in the treatment of chronic diseases. At the same time, we suggest that rheumatologists should proactively inform SS patients during routine treatment about the insidious risk of osteoporosis. Given the high and rising burden of osteoporosis, healthcare providers should routinely assess bone function and institute novel and safe approaches for disease management for SS persons, including uses of complementary and alternative therapies.

## Figures and Tables

**Table 1:** Demographic data and selected comorbidities of cases and controls.

Variables	Number (%)	Cases	Controls	Standardized Difference
		N = 1240 (%)	N = 1240(%)	
Age (Years)				0.001
≤50	1286 (51.9)	641 (51.7)	645 (52.0)	
>50	1194 (48.1)	599 (48.3)	595 (48.0)	
Mean (SD)	48.5 (14.0)	48.2 (14.1)	48.9 (14.0)	0.004
Gender				0.003
Male	497 (20.0)	255 (20.6)	242 (19.5)	
Female	1983 (80.0)	985 (79.4)	998 (80.5)	
Monthly income				0.001
25th percentile	1214 (49.0)	606 (48.9)	608 (49.0)	
50th percentile	1130 (45.6)	573 (46.2)	557 (44.9)	
75th percentile	136 (5.5)	61 (4.9)	75 (6.0)	
Residential typologies				0.04
Urban area	1506 (60.7)	737 (59.4)	769 (62.0)	
Suburban area	399 (16.1)	209 (16.9)	190 (15.3)	
Rural area	575 (23.2)	294 (23.7)	281 (22.7)	
CCI	2.7 (2.6)	2.7 (2.3)	2.6 (2.8)	0.03
Anti-osteoporotic medications	1158 (46.7)	586 (47.3)	572 (46.1)	0.04

**Table 2:** Relationship between use of CHM and subsequent risk of osteoporosis.

CHM Exposure	Subjects				Crude OR (95% CI)	Adjusted OR * (95% CI)
	Cases n = 1240		Controls n = 1240			
Non-CHM Users	886	71.5%	582	46.9%	1	1
CHM users	334	28.5%	658	53.1%	0.49 (0.41–0.57)	0.47 (0.39–0.57)
Level 1 (31–180 days)	280	22.6%	445	35.9%	0.56 (0.46–0.66)	0.56 (0.45–0.67)
Level 2 (181–365 days)	63	5.1%	160	12.9%	0.35 (0.26–0.46)	0.34 (0.24–0.47)
Level 3 (366 days or more)	11	0.9%	53	4.3%	0.32 (0.19–0.55)	0.29 (0.17–0.49)

* Adjusted for potential confounders that included age, gender, residential area, monthly income and CCI.

**Table 3:** Age- and gender-specific risk of osteoporosis among SS patients with and without CHM use.

Variables	Osteoporosis Cases, n (%)	Crude OR (95% CI)	Adjusted OR (95% CI)
Gender			
Male	255 (20.6)	0.52 (0.35–0.76)	0.53 (0.37–0.79) *
Female	985 (79.4)	0.32 (0.27–0.36)	0.30 (0.24–0.36) *
Age (years)			
≤50	641 (49.8)	0.31 (0.25–0.40)	0.31 (0.25–0.38) **
>50	599 (50.2)	0.39 (0.37–0.50)	0.37 (0.31–0.52) **

* Model adjusted for age, residential area, monthly income, and CCI. ** Model adjusted for gender, residential area, monthly income, and CCI.

## Data Availability

This study obtained data from the National Health Insurance Research Database provided by the Bureau of National Health Insurance, managed by the Department of Health and Welfare, Taiwan. Because of restrictions imposed by the law of “Personal Information Protection Act”, the relevant data cannot be publicly obtained. Requisition for usage of datasets should be directed to the Bureau of National Health Insurance and the corresponding author.

## References

[1] Thurtle E., Grosjean A., Steenackers M., Strege K., Barcelos G., Goswami P. (2023). Epidemiology of Sjögren’s: A systematic literature review. Rheumatol. Ther..

[2] Qin B., Wang J., Yang Z., Yang M., Ma N., Huang F., Zhong R. (2015). Epidemiology of primary Sjögren’s syndrome: A systematic review and meta-analysis. Ann. Rheum. Dis..

[3] Perera S., Ma L., Punwaney R., Ramachandran S. (2018). Clinical and Cost Burden of Primary Sjögren’s Syndrome: Descriptive Analysis Using a US Administrative Claims Database. J. Health Econ. Outcomes Res..

[4] Skarlis C., Palli E., Nezos A., Koutsilieris M., Mavragani C.P. (2018). Study of the incidence of osteoporosis in patients with Sjögren’s syndrome (pSS) and investigation of activation of the RANKL/RANK and osteoprotegerin (OPG) system. Mediterr. J. Rheumatol..

[5] Benchabane S., Boudjelida A., Toumi R., Belguendouz H., Youinou P., Touil-Boukoffa C. (2016). A case for IL-6, IL-17A, and nitric oxide in the pathophysiology of Sjögren’s syndrome. Int. J. Immunopathol. Pharmacol..

[6] Luo J., Ming B., Zhang C., Deng X., Li P., Wei Z., Xia Y., Jiang K., Ye H., Ma W. (2018). IL-2 Inhibition of Th17 Generation Rather Than Induction of Treg Cells Is Impaired in Primary Sjögren’s Syndrome Patients. Front. Immunol..

[7] Weitzmann M.N. (2017). Bone and the immune system. Toxicol. Pathol..

[8] Jimi E., Takakura N., Hiura F., Nakamura I., Hirata-Tsuchiya S. (2019). The role of NF-κB in physiological bone development and inflammatory bone diseases: Is NF-κB inhibition “Killing two birds with one stone”?. Cells.

[9] Kuo P.I., Lin T.M., Chang Y.S., Hou T.Y., Hsu H.C., Lin S.H., Chen W.S., Lin Y.C., Wang L.H., Chang C.C. (2021). Primary Sjogren syndrome increases the risk of bisphosphonate-related osteonecrosis of the jaw. Sci. Rep..

[10] Kanis J.A., Oden A., Johnell O., De Laet C., Jonsson B., Oglesby A.K. (2003). The components of excess mortality after hip fracture. Bone.

[11] Luo H., Li X., Liu J., Andrew F., George L. (2012). Chinese Herbal medicine in treating primary sjögren’s syndrome: A systematic review of randomized trials. Evid.-Based Complement. Altern. Med. Ecam.

[12] Chen H.H., Lai J.N., Yu M.C., Chen C.Y., Hsieh Y.T., Hsu Y.F., Wei J.C. (2021). Traditional Chinese medicine in patients with primary sjogren’s syndrome: A randomized, double-blind, placebo-controlled clinical trial. Front. Med..

[13] Chang C.M., Wu P.C., Lin J.R., Jan Wu Y.J., Luo S.F., Hsue Y.T., Lan J.L., Pan T.L., Wu Y.T., Yu K.H. (2021). Herbal formula SS-1 increases tear secretion for sjögren’s syndrome. Front. Pharmacol..

[14] Doss H.M., Samarpita S., Ganesan R., Rasool M. (2018). Ferulic acid, a dietary polyphenol suppresses osteoclast differentiation and bone erosion via the inhibition of RANKL dependent NF-κB signalling pathway. Life Sci..

[15] Cheng C.F., Lin Y.J., Tsai F.J., Li T.M., Lin T.H., Liao C.C., Huang S.M., Liu X., Li M.J., Ban B. (2019). Effects of Chinese herbal medicines on the risk of overall mortality, readmission, and reoperation in hip fracture patients. Front. Pharmacol..

[16] Shih C.C., Liao C.C., Su Y.C., Tsai C.C., Lin J.G. (2012). Gender differences in traditional Chinese medicine use among adults in Taiwan. PLoS ONE.

[17] Aljefree N.M., Almoraie N.M., Althaiban M.A., Hanbazaza M.A., Wazzan H.A., Shatwan I.M. (2023). Gender differences in knowledge, attitudes, and practices with respect to type 1 diabetes among Saudi public-school teachers. BMC Public Health.

[18] Ikeda K., Horie-Inoue K., Inoue S. (2019). Functions of estrogen and estrogen receptor signaling on skeletal muscle. J. Steroid Biochem. Mol. Biol..

[19] Minniti G., Pescinini-Salzedas L.M., Minniti G.A.D.S., Laurindo L.F., Barbalho S.M., Vargas Sinatora R., Sloan L.A., Haber R.S., AraAojo A.C., Quesada K. (2022). Organokines, sarcopenia, and metabolic repercussions: The vicious cycle and the interplay with exercise. Int. J. Mol. Sci..

[20] Kim J.H., Kim M., Hong S., Kwon B., Song M.W., Song K., Kim E.Y., Jung H.S., Sohn Y. (2021). Anti-inflammatory effects of Fritillaria thunbergii Miquel extracts in LPS-stimulated murine macrophage RAW 264.7 cells. Exp. Ther. Med..

[21] Laksmitawati D.R., Widyastuti A., Karami N., Afifah E., Rihibiha D.D., Nufus H., Widowati W. (2017). Anti-inflammatory effects of Anredera cordifolia and Piper crocatum extracts on lipopolysaccharide-stimulated macrophage cell line. Bangladesh J. Pharmacol..

[22] Hu W., Yang X., Zhe C., Zhang Q., Sun L., Cao K. (2011). Puerarin inhibits iNOS, COX-2 and CRP expression via suppression of NF-κB activation in LPS-induced RAW264.7 macrophage cells. Pharmacol. Rep. PR.

[23] An S., Han F., Hu Y., Liu Y., Li J., Wang L. (2018). Curcumin inhibits polyethylene-induced osteolysis via repressing NF-κB signaling pathway activation. Cell Physiol. Biochem..

[24] Shrimali D., Shanmugam M.K., Kumar A.P., Zhang J., Tan B.K., Ahn K.S., Sethi G. (2013). Targeted abrogation of diverse signal transduction cascades by emodin for the treatment of inflammatory disorders and cancer. Cancer Lett..

[25] Ding D., Yan J., Feng G., Zhou Y., Ma L., Jin Q. (2022). Dihydroartemisinin attenuates osteoclast formation and bone resorption via inhibiting the NF-κB, MAPK and NFATc1 signaling pathways and alleviates osteoarthritis. Int. J. Mol. Med..

[26] Sharma A.R., Jagga S., Chakraborty C., Lee S.-S. (2020). Fibroblast-like-synoviocytes mediate secretion of pro-inflammatory cytokines via ERK and JNK MAPKs in ti-particle-induced osteolysis. Materials.

[27] Tsai F.J., Ho T.J., Cheng C.F., Shiao Y.T., Chien W.K., Chen J.H., Liu X., Tsang H., Lin T.H., Liao C.C. (2017). Characteristics of Chinese herbal medicine usage in ischemic heart disease patients among type 2 diabetes and their protection against hydrogen peroxide-mediated apoptosis in H9C2 cardiomyoblasts. Oncotarget.

[28] Cortés-Vieyra R., Silva-García O., Gómez-García A., Gutiérrez-Castellanos S., Álvarez-Aguilar C., Baizabal-Aguirre V.M. (2021). Glycogen synthase kinase 3β modulates the inflammatory response activated by bacteria, viruses, and parasites. Front. Immunol..

[29] Yasui T., Yamada M., Uemura H., Ueno S.-i., Numata S., Ohmori T., Tsuchiya N., Noguchi M., Yuzurihara M., Kase Y. (2009). Changes in circulating cytokine levels in midlife women with psychological symptoms with selective serotonin reuptake inhibitor and Japanese traditional medicine. Maturitas.

[30] Guo R.B., Wang G.F., Zhao A.P., Gu J., Sun X.L., Hu G. (2012). Paeoniflorin protects against ischemia-induced brain damages in rats via inhibiting MAPKs/NF-κB-mediated inflammatory responses. PLoS ONE.

[31] Inagaki Y., Kido J.-i., Nishikawa Y., Kido R., Sakamoto E., Bando M., Naruishi K., Nagata T., Yumoto H. (2021). Gan-lu-yin (kanroin), traditional Chinese herbal extracts, reduces osteoclast differentiation in vitro and prevents alveolar bone resorption in rat experimental periodontitis. J. Clin. Med..

[32] Qu Z., Zhang B., Kong L., Gong Y., Feng M., Gao X., Wang D., Yan L. (2022). Receptor activator of nuclear factor-κB ligand-mediated osteoclastogenesis signaling pathway and related therapeutic natural compounds. Front. Pharmacol..

[33] Liu T., Zhang L., Joo D., Sun S.C. (2017). NF-κB signaling in inflammation. Signal Transduct. Target. Ther..

[34] National Health Insurance Database, Taiwan LHID 2000. Taiwan: Center for Biomedical Resources of NHRI; 2012. https://nhird.nhri.edu.tw//en/index.html.

[35] Huang S.C., Gau S.Y., Huang J.Y., Wu W.J., Wei J.C. (2022). Increased risk of hypothyroidism in people with asthma: Evidence from a real-world population-based study. J. Clin. Med..

[36] Liu C.Y., Hung Y.T., Chuang Y.L., Chen Y.J., Weng W.S., Liu J.S., Liang K.Y. (2006). Incorporating development stratification of Taiwan townships into sampling design of large scale health interview survey. J. Health Manag..

[37] Deyo R.A., Cherkin D.C., Ciol M.A. (1992). Adapting a clinical comorbidity index for use with ICD-9-CM administrative databases. J. Clin. Epidemiol..

[38] Austin P.C. (2008). A critical appraisal of propensity-score matching in the medical literature between 1996 and 2003. Stat. Med..

